# Early outcomes of preterm neonates with respiratory distress syndrome admitted at Muhimbili National Hospital, a prospective study

**DOI:** 10.1186/s12887-022-03731-2

**Published:** 2022-12-22

**Authors:** Maria Bulimba, Judith Cosmas, Yaser Abdallah, Augustine Massawe, Karim Manji

**Affiliations:** 1 Department of Paediatrics and child health, Muhimbili University of Health and Allied Sciences, P.O BOX 65001 Dar es Salaam, Tanzania; 2 Department of Paediatrics and Child Health, The Aga Khan Hospital, P.O BOX 2289 Dar es Salaam, Tanzania; 3 Department of Paediatrics and Child Health, Muhimbili National Hospital, P.O BOX 65000 Dar es Salaam, Tanzania

**Keywords:** Prematurity, Respiratory distress syndrome, Mortality

## Abstract

**Background:**

Respiratory distress syndrome (RDS) is one of the commonest complication preterm neonates suffer and accounts for a significant morbidity and mortality in low and middle income countries (LMICs). Addressing RDS is therefore crucial in reducing the under 5 mortality in LMICs. This study aimed at describing early outcomes (death/survival) of preterm neonates with RDS and identify factors associated with the outcomes among neonates admitted at Muhimbili national hospital, Tanzania.

**Methods:**

Between October 2019 and January 2020 we conducted a prospective study on 246 preterm neonates with RDS at Muhimbili National Hospital. These were followed up for 7 days. We generated Kaplan–Meier survival curve to demonstrate time to death. We performed a cox regression analysis to ascertain factors associated with outcomes. The risk of mortality was analyzed and presented with hazard ratio. Confidence interval of 95% and *P*-value less than 0.05 were considered as significant.

**Results:**

Of the 246 study participants 51.6% were male. The median birth weight and gestational age of participants (Inter-Quartile range) was 1.3 kg (1.0, 1.7) and 31 weeks (29, 32) respectively. Majority (60%) of study participants were inborn. Only 11.4% of mothers of study participants received steroids. Of the study participants 49 (20%) received surfactant. By day 7 of age 77/246 (31.3%) study participants had died while the majority of those alive 109/169 (64.5%) continued to need some respiratory support. Factors independently associated with mortality by day 7 included birth weight of < 1500 g (AHR = 2.11 (1.16–3.85), CI95%; *p* = 0.015), lack of antenatal steroids (AHR = 4.59 (1.11–18.9), CI95%; *p* = 0.035), 5th minute APGAR score of < 7 (AHR = 2.18 (1.33–3.56), CI95%; *p* = 0.002) and oxygen saturation < 90% at 6 hours post admission (AHR = 4.45 (1.68–11.7), CI95%; *p* = 0.003).

**Conclusion:**

Our study reports that there was high mortality among preterm neonates admitted with RDS mainly occurring within the first week of life. Preterm neonates with very low birth weight (VLBW), whose mother did not receive antenatal steroid, who scored < 7 at 5th minute and whose saturation was < 90% at 6 hours were at higher risk of dying. There is need to scale up antenatal corticosteroids, neonatal resuscitation training and saturation monitoring among preterm neonates with RDS.

## Background

Respiratory distress syndrome (RDS) is among the major complications of preterm birth [1]. The risk of RDS is related to production of surfactant in the lungs. RDS increases with degree of prematurity and its frequency decreases significantly after 37 weeks of gestation [2].

The clinical signs of RDS manifest within the first minutes or hours after birth and progress over the first 48 to 72 hours thereafter, begins to resolve. The subsequent improvement coincides with increased production of endogenous surfactant resulting in resolution of symptoms by one week of age [3].

The diagnosis of RDS is based on clinical findings of a preterm infant with onset of progressive respiratory distress shortly after birth and a characteristic chest radiograph, which demonstrates low lung volume and diffuse reticulogranular ground glass appearance with air bronchograms [4].

The management of RDS has evolved over the past few decades in the developed countries [5, 6]. Despite the increase in RDS cases, in developed countries the infant mortality rate from RDS has significantly reduced but in resource restricted setting RDS is still among the commonest cause of mortality among preterm neonates [5, 7].

The findings of previous studies in the globe revealed that several factors are associated with mortality in preterm neonates with RDS. These include: gestational age [8–12], birth weight [9–12], sex [13, 14], APGAR score [15, 16], hypothermia [17], Silverman Andersen score (SAS) [10], place of delivery [12, 18], Antenatal Corticosteroids (ACS) [8, 11, 19–21], mode of delivery [11, 12], Continuous positive airway pressure (CPAP) [22–25], and surfactant [23].

Despite interventions recommended by WHO to improve outcome of preterm neonates, mortality of preterm neonates in low- and middle-income countries remains significant. Tanzania is among countries with high preterm births and hence of RDS. Neonatal mortality contributes a significant proportion to under 5 deaths. We conducted this study to ascertain outcome of preterm neonates admitted with RDS and identifying factors associated with the outcomes. This knowledge is important in identifying area needing improvement to reduce preterm neonatal deaths and hence under 5 deaths.

## Methods

### Setting

A prospective study was conducted at the newborn intensive care unit (NICU) of Muhimbili National Hospital (MNH), Dar-es-Salaam, Tanzania. MNH is a national tertiary level referral hospital for Tanzania and a teaching hospital for Muhimbili University of Health and Allied Sciences. Neonatal unit is located at the maternity block, second floor, it has two wards one for preterm (ward 37) and another for term babies (ward 36). In preterm ward there are 4 cubes, with a total bed capacity of 60 beds. At the time of the study the unit has wall mounted oxygen, 3 mechanical ventilators, 5 radiant warmers, 5 wall cardiac monitors, 5 diamedic continuous positive airway pressure (CPAP), 10 high flow CPAP and 20 infusion pumps. It has well trained staffs for premature care, 7 specialist, 3 registered medical officers, 3–5 Paediatric resident’s, minimum of 4 intern medical doctors and well-trained nurses. MNH has approximately 10,000 neonates born per year both term and preterm. Admissions in preterm unit is approximately 4–10 babies per day with monthly admission rate of 120–280 neonates and an annual admission rate of 1440–3360 neonates, of which RDS is one of the admission diagnoses. MNH neonatal unit therefore was selected purposively because most preterm infants with RDS are admitted here.

Inborn preterm neonates are received by midwives in the delivery rooms and transferred immediately to NICU. Delayed cord clamping is hardly observed for preterm infants and no CPAP is provided in the delivery rooms.

Preterm neonates receive intravenous fluids, antibiotics, and aminophylline. They are cared for on open radiant warmers with on many occasions one warmer hosting 2-3infants. Preterm neonates with SAS score of > 3 are commenced on CPAP with blended oxygen but not with heated air. Infants needing < 30% oxygen with SAS score < 3 are transitioned to nasal prong oxygen 2 l/min and gradually weaned off. Surfactant is given to infants with severe distress whose parents can purchase.

Tanzania is a lower-middle-income country, it has an estimated population of about 60 million people. Health services in the United Republic of Tanzania are delivered through a decentralized system. Access to health care in Tanzania is still a challenge especially in women. Health insurance coverage is still low approximately 32%.

### Study population

All preterm neonates < 24 hours old with clinical signs of RDS. Clinical signs of RDS which starts in < 6 hours after birth were tachypnea, nasal flaring, expiratory grunting, intercostal, subcostal, and subxiphoid retractions, and cyanosis.

### Variables of study

#### Outcome variables

The outcome for this study was Death or Survival at 7 days of life.

#### Independent variables

In this study, maternal and Preterm neonates variables were considered. Antenatal steroid, Place of delivery, Mode of delivery, pPROM> 18 hours, maternal fever. Gestational age, Birth weight, Sex, APGAR score at 5th minute, Age at admission, SAS at admission and 6 hours, Oxygen saturation at commence of care and at 6 hours, Body temperature, random blood sugar, Surfactant.

### Procedure

Between October 2019 and January 2020, all preterm neonates < 24 hours of age whose respiratory distress commenced < 6 hours after birth were enrolled. New Ballard score [26] was used to determine gestational age and then preterm neonates were categorized in two groups for analysis (< 32 weeks and > 32 weeks), while Silverman Andersen score (SAS) [27] was used to grade the severity of RDS. Neonates with major anomalies and those with severe birth asphyxia (APGAR score < 4 at 5 min) were excluded.

All study variables were obtained from maternal history, antenatal cards and NICU records. These were entered in the data sheet.

Daily follow-up was done, vital signs and oxygen saturations were observed daily or obtained from the nursing charts.

### Data management and analysis

The data was analysed using SPSS software packages version 23.0. Frequency distribution and Kaplan–Meier survival curves were used to show pattern of death in 7 days for birth weight and gestational age. Independent and adjusted relationships of different predictors with preterm neonates’ survival were assessed with Cox regression model. Factors associated with mortality in crude model with a *p* value < 0.05 were entered in the multivariable Cox regression model to identify and quantify predictors of deaths while controlling for potential confounder. The risk of mortality was explored and presented with hazard ratio and 95% confidence interval. *P*-value less than 0.05 was considered as significant.

## Results

The median birth weight of participants (Inter-Quartile range) was 1.3 (1.0, 1.7) kilograms. Of the study participants 79/246 (32%) were small for gestational age (SGA). The median gestational age (Inter-Quartile range) was 31 (29, 32) weeks. The median age of participants (Inter-Quartile range) was 1.3 (0.7, 5.5) hours at admission and median age at start of respiratory support (Inter-Quartile range) was 1.66 (0.75, 6.41) hours.

Most 240 (97.6%) preterm neonates presented with respiratory rate > 60breath/minute and nasal flaring at recruitment. Other clinical presentation were subcostal retractions 202 (82.1%) and grunting 131 (53.3%). (Table 1).

**Table 1:** Demographic and baseline characteristics of study participants

***Characteristics***	***N (%)***
**Sex**	
Male	127 (51.6)
Female	119 (48.4)
**Gestational Age (weeks)**	
26 to < 28	9 (3.7)
28 to ≤32	132 (53.7)
> 32 to 37	105 (42.7)
**SGA**	
Yes	79 (32.1)
No	167 (67.9)
**Birth weight (grams)**	
< 1500	152 (61.8)
≥ 1500	94 (38.2)
**5th minute APGAR Score**	
> 7	156 (63.4)
4–7	80 (32.8)
Not known	10 (3.8)
**Age at admission**	
< 1 hour	89 (36.2)
≥ 1 hour-24 hours	157 (63.8)
**Age at start of respiratory support**	
< 1 hour	77 (31.3)
≥ 1 hour	169 (68.7)
**Respiratory support initiated**	
CPAP	158 (64.2)
Oxygen by Nasal prongs	82 (33.3)
Mechanical ventilation	6 (2.4)
**Surfactant**	
Yes	49 (19.9)
No	197 (80.1)
**Maternal Age**	
< 20 years	21 (8.5)
20–34 years	175 (71.2)
> 35 years	50 (20.3)
**Parity**	
Primipara	79 (32.1)
Multipara	167 (67.9)
**Antenatal steroid (Dexamethasone)**	
Received	28 (11.4)
Not received	218 (88.6)
**Prolonged rapture of membranes > 18 hours (PROM)**	
Yes	82 (33.3)
No	164 (66.7)
**Maternal Fever**	
Yes	33 (13.4)
No	213 (86.6)
**Maternal Diabetes**	
Yes	2 (0.8)
No	244 (99.2)

At 6 hours of admission the majority 56.1% had moderate to severe respiratory distress, a third 32.1% were hypothermic and 11.4% were hypoglycemic (Table 2).

**Table 2:** Vitals at admission and at 6 hours after admission

***Characteristics***	***At admission N (%)***	***At 6 hours N (%)***
**Silverman Andersen score**		
≥ 6 (severe)	66 (26.8)	50 (20.3)
4 to 5 (moderate)	83 (33.7)	88 (35.8)
1 to 3 (mild)	97 (39.4)	108 (43.9)
**Oxygen Saturation**		
≥ 90%	156 (63.4)	238 (96.7)
< 90%	90 (36.6)	8 (3.3)
**Temperature (°c)**		
**≥ 36.5**	109 (44.3)	167 (67.9)
**< 36.5**	137 (55.7)	79 (32.1)
**Random blood sugar (mmol/L)**		
**≥ 2.6**	172 (69.9)	218 (88.6)
**< 2.6**	74 (30.1)	28 (11.4)

The overall mortality was 31.3% there as 77/246, 35 (45.5%) deaths occurred in the first 72 hours. Mortality among preterm neonates with birth weight < 1500 g was 40% and those with birth weight ≥ 1500 g was 17%. Among preterm neonates who were alive in the first week of life, 109 (64.5%) were still in need of respiratory support.

**Fig. 1:**
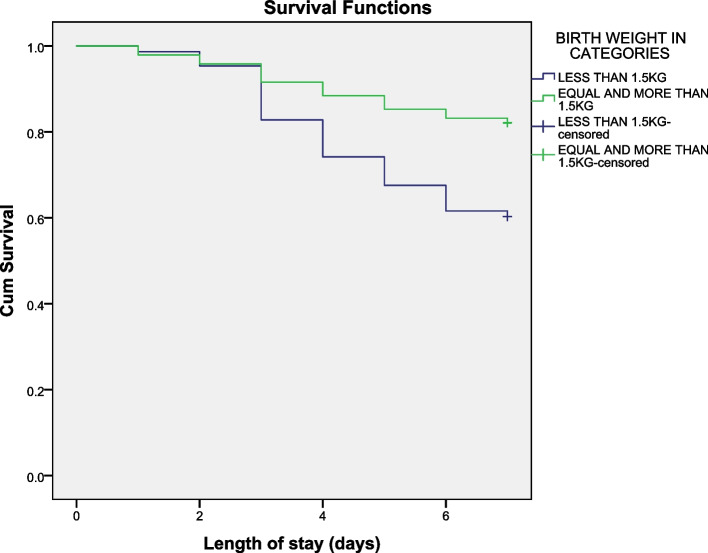
Kaplan-Meier survival pattern of Preterm Neonates and Birth weight

**Fig. 2:**
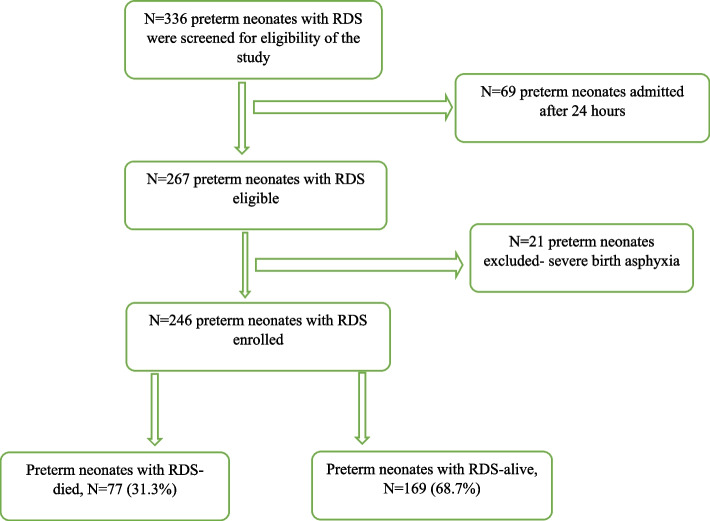
Patient Recruitment

**Fig. 3:**
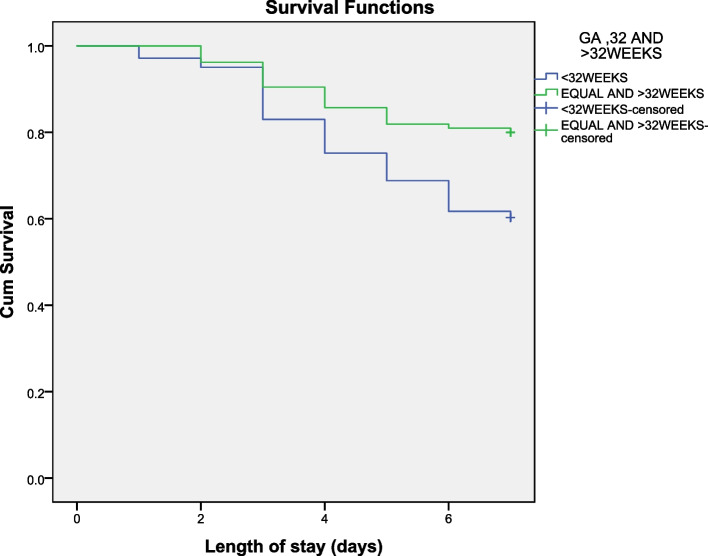
Kaplan-Meier survival pattern of Preterm Neonates and Gestational Age

From the log rank test, survival distribution of different birth weight categories was statistically significant (P = < 0.001) indicating that those with birth weight ≥ 1500 g were more likely to survive (Fig. 1). From the log rank test, survival distribution of different GA categories was statistically significant (*P* = 0.002) indicating that having a GA above 32 weeks was significantly associated with better survival (Fig. 2). On day 7, the probability of survival of preterm neonate who had GA above 32 weeks was 80% and for a preterm neonate who had GA < 32 weeks was 60% (Fig. 3).

### Cox proportional hazards regression models

Variables with *P*-value less than 0.05 in crude model were included in the Cox proportional hazards regression model. Gestational age, birth weight, Apgar score at 5th minute, age at admission, temperature at 6 hours of admission, antenatal steroids, oxygen saturation at admission and at 6 hours post admission were variables included in multivariate Cox regression model. After adjusting for confounders, we found that hazards of preterm mortality by day 7 of age was high for preterm neonates with birth weight < 1500 g (AHR = 2.11 (1.16–3.85), CI95%; *p* = 0.015), preterms whose mother’s lack antenatal steroids (AHR = 4.59 (1.11–18.9), CI95%; *p* = 0.035), preterm neonates with APGAR score at 5th minute of 4–7 (AHR = 2.18 (1.33–3.56), CI95%; *p* = 0.002) and preterm neonates with oxygen saturation < 90% at 6 hours post admission (AHR = 4.45 (1.68–11.7), CI95%; *p* = 0.003), (Table 3).

**Table 3:** Determinants of Survival in Preterm neonates with Respiratory distress syndrome

***Factors***	***Preterm Survival Status***					
	Censored N (%)	Died N (%)	CHR 95% CI	***P-value***	AHR 95% CI	***P-value***
**Sex**						
Male	93 (73.2)	34 (26.8)	1.45 (0.92–2.27)	0.11		
Female	76 (63.9)	43 (36.1)	1		–	–
**Gestational Age**						
< 32 weeks	85 (60.3)	56 (39.7)	2.16 (1.31–3.56)	0.003*	1.61 (0.91–2.85)	0.10
≥ 32–37 weeks	84 (80)	21 (20)	1		1	
**Birth weight (grams)**						
< 1500	91 (59.9)	61 (40.1)	2.45 (1.43–4.20)	0.001*	2.11 (1.16–3.85)	0.015*
≥ 1500	78 (83)	16 (17)	1		1	
**5th minute APGAR Score**						
> 7	116 (74.4)	40 (25.6)	1		1	
4–7	48 (60)	32 (40)	1.71 (1.07–2.72)	0.024*	2.18 (1.33–3.56)	0.002*
Not known	5 (50)	5 (50)	2.22 (0.87–5.61)	0.09	1.97 (0.74–5.21)	0.17
**Age at admission**						
< 1 hour	69 (77.5)	20 (22.5)	1		1	
≥ 1 hour	100 (63.7)	57 (36.3)	1.73 (1.04–2.88)	0.036*	1.56 (0.91–2.67)	0.10
**Admission saturation**						
≥ 90%	115 (73.7)	41 (26.3)	1		1	
< 90%	54 (60)	36 (40)	1.69 (1.08–2.64)	0.022*	1.50 (0.95–2.38)	0.09
**Admission SAS**						
≥ 6 (severe)	50 (75.8)	16 (24.2)	0.79 (0.43–1.46)	0.46		
4 to 5 (moderate)	51 (61.4)	32 (38.6)	1.36 (0.82–2.25)	0.23		
1 to 3 (mild)	68 (70.1)	29 (29.9)	1		–	–
**Admission temp (°C)**						
≥ 36.5	81 (74.3)	28 (25.7)	1		–	–
< 36.5	88 (64.2)	49 (35.8)	1.46 (0.92–2.32)	0.11		
**Admission RBS**						
≥ 2.6 mmol/l	124 (72)	48 (28)	1		–	–
< 2.6 mmol/l	45 (61)	29 (39)	1.54 (0.97–2.43)	0.07		
**6th hour temp (°C)**						
≥ 36.5	123 (73.7)	44 (26.3)	1		1	
< 36.5	46 (58.2)	33 (41.8)	1.67 (1.07–2.63)	0.025*	1.02 (0.63–1.66)	0.92
**6Th hour RBS**						
≥ 2.6 mmol/l	151 (69.3)	67 (30.7)	1		–	–
< 2.6 mmol/l	18 (64.3)	10 (35.7)	1.23 (0.63–2.38)	0.55		
**6th hour saturation**						
≥ 90%	166 (69.7)	72 (30.3)	1		1	**–**
< 90%	3 (37.5)	5 (62.5)	3.03 (1.22–7.52)	0.017*	4.45 (1.68–11.7)	0.003*
**6th hour SAS**						
≥ 6 (severe)	37 (74)	13 (26)	0.87 (0.46–1.67)	0.68		
4–5 (moderate)	56 (63.6)	32 (36.4)	1.25 (0.76–2.04)	0.38		
1–3 (mild)	76 (70.4)	32 (29.6)	1		–	–
**Surfactant**						
Yes	38 (77.6)	11 (22.4)	1		–	–
No	131 (66.5)	66 (33.5)	1.61 (0.85–3.0)	0.15		
**Place of Delivery**						
MNH	109 (73.2)	40 (26.8)	1		–	–
Others	60 (62)	37 (38)	1.47 (0.94–2.31)	0.09		
**Mode of Delivery**						
Vaginal	108 (66.7)	54 (33.3)	1		–	–
Caesarian Section	61 (2.6)	23 (27.4)	0.82 (0.50–1.34)	0.43		
**Antenatal steroid**						
Received	26 (92.9)	2 (7.1)	1		1	
Not received	143 (65.6)	75 (34.4)	5.35 (1.31–21.8)	0.019*	4.59 (1.11–18.9)	0.035*
**PROM > 18 hours**						
Yes	51 (62.2)	31 (37.8)	1.38 (0.88–2.18)	0.17		
No	118 (72)	46 (28)	1		–	**–**
**Maternal Fever**						
Yes	20 (60.6)	13 (39.4)	1.42 (0.78–2.58)	0.25		
No	149 (70)	64 (30)	1		–	–

* = significant at *P* < 0.05, *CHR* Crude Hazard ratio, *AHR* Adjusted Hazard ratio, *95% CI  *95% Confidence Interval

## Discussion

This study aimed to determine the early outcomes and associated factors among preterm neonates with RDS admitted at MNH. Results from this study shows that the mortality rate during the first week of life was 31.3% and Preterm neonates with very low birth weight (VLBW), whose mother did not receive antenatal steroid, who scored APGAR < 7 at 5th minute and whose oxygen saturation was < 90% at 6 hours were at higher risk of dying.

The mortality rate of preterm neonates with RDS have a wide variation ranging from 6.5 to 88% [22, 28–30]. Our findings are consistent with several studies in Low Income Countries [30, 31]. A previous study in the same unit showed higher rates of mortality in premature babies due to RDS mostly occurring in the first week of life 88% and another study in a different region was 81.1% [28, 32]. Comparing with this current study the mortality has reduced. This is possibly because of the introduction of CPAP [33] as other supporting care has been the same except earlier on there was no easy availability of CPAP.

In this study birth weight was found to be independently associated with outcome death. Mortality was higher among neonates with weight < 1500 g (40%) compared to those > 1500 g (17%), this might be because those < 1500 g were more premature and had more severe disease than those > 1500 g [2].

We observed that preterm neonates with low oxygen saturation < 90% were at higher risk for mortality (AHR = 4.45 (1.68–11.7), CI95%; *p* = 0.003). This is similar to one study done by Sathenahalli et al. [12] Infants with RDS who fail to maintain saturation were likely in respiratory failure. Low oxygen saturation target has previously been demonstrated to be associated with high mortality [34].

Infants with RDS and Apgar score at 5th minute of 4–7 had higher mortality rate of 40% compared to those with Apgar score at 5th minute of more than 7 which was 25.6%. Thus, low Apgar score at 5th minute was an independent predictor of mortality. This finding is comparable to other two studies which showed increase in RDS and mortality in preterm babies with low Apgar score [15, 16]. This may be because of the following two reasons, one is acute lung injury caused by severe birth asphyxia decreases the synthesis and secretion of pulmonary surfactant and two hypoxia which inhibits the activity of surfactant and even leads to its inactivation. Moreover, severe asphyxia can increase mortality and thus, it is important for us to tailor high impact maternal and preterm care interventions to reduce mortality [35].

Only 2/28 (7.1%) study participants whose mother received steroids died and 75/200 (34.4%) of those whose mothers did not receive steroids died. These findings were comparable with a study done in India by Palod P. H et al. [11]. Similar observation from other studies have also shown that lack of antenatal steroids were associated with higher mortality rates in premature babies with RDS [8, 21, 36, 37]. This can be explained as steroids help in lung maturation especially in extreme to very preterm babies, and thus improving lung function. Also contributes to less severe disease as it has been shown that use of antenatal steroids reduces the incidence of RDS and mortality in preterm neonates [38]. Again, Initiative of implementing the care bundle which was done in Tanzania in four hospitals, including MNH for preterm infants was associated with significant reduction in neonatal mortality presumably by reducing respiratory morbidity with antenatal steroids and minimizing infection with antibiotics [21]. In this current study only 11.4% received antenatal steroids this might be due to gaps in implementation of evidence-based guideline at various levels, poor identification and follow up of women at risk of preterm delivery and high level of spontaneous preterm deliveries in otherwise normal pregnancies.

Preterm neonates who received surfactant had less mortality; however, it was not an independent predictor of mortality. These finding are similar to the study done by Niknafs et al. [25] which showed no difference in outcome between two groups of surfactants. In contrast to other study done by Hala et al. [23] showed a significant reduction in mortality by 50% with administration of timely surfactant. In current study there was slight decrease in mortality among the 20% preterm neonates who received surfactant this might be due to majority received surfactant late but also cost implication which required parental consent.

We still observe high mortality in our setting (LMIC) as compared to high income countries. Our setting is equipped but overwhelmed by number of premature babies delivered with complications, and thus compromising quality of care and availability of resources. Low uptake of ACS as demonstrated from our findings it talks about our poor health care access as well as lack of surfactant due to cost implication which parents need to cover while most of our mothers can’t afford.

### Study limitations

Lack of chest radiography to confirm the diagnosis, severity, and possible complications such as air leaks was lacking, and this would affect the interpretation of some findings.

All preterm neonates are managed empirically with antibiotics for presumed sepsis without appropriate investigations hence difficulty to delineate between sepsis and RDS.

Lack of routine cranial scan on these vulnerable infants made it difficult to ascertain other contributors to poor outcome including death.

## Conclusion

Mortality of premature neonates with respiratory distress syndrome (RDS) admitted at MNH was 31.3% in the first week of life. Preterm neonates with very low birth weight (VLBW), whose mother did not receive antenatal steroid, who scored < 7 at 5 min and whose saturation was < 90% at 6 hours were at higher risk of dying.

Tailored interventions addressing risk factors should be devised to improve the preterm neonate’s survival in the study area. Close attention to early stabilization and improve preterm care may further reduce the mortality associated with this disorder especially to those with low birth weight and lower gestational age.

## Data Availability

The data from this study can be obtained upon request from the first author, Dr. Maria Bulimba email: mbulimba@yahoo.com.

## Abbreviations

AHR: Adjusted Hazard Ratio
CPAP: Continuous Positive Airway Pressure
CHR: Crude Hazard Ratio
GA: Gestational Age
GCP: Good clinical practice
LMIC: Low middle income countries
MNH: Muhimbili National Hospital
MUHAS: Muhimbili University of Health and Allied Sciences
NICU: Neonatal Intensive Care Unit
PROM: Premature Rupture of Membrane
RBS: Random Blood Sugar
RDS: Respiratory Distress Syndrome
SAS: Silverman Andersen score
SGA: Small for Gestational Age
VLBW: Very low birth weight
